# Defect-induced nucleation and epitaxial growth of a MOF-derived hierarchical Mo_2_C@Co architecture for an efficient hydrogen evolution reaction†

**DOI:** 10.1039/d0ra01197e

**Published:** 2020-04-06

**Authors:** Linfei Zhang, Jingting Zhu, Yumeng Shi, Zhuo Wang, Wenjing Zhang

**Affiliations:** Institute of Microscale Optoelectronics, Shenzhen University Shenzhen 518060 P. R. China wjzhang@szu.edu.cn wzhuo@szu.edu.cn; College of Physics and Optoelectronic Engineering, Shenzhen University Shenzhen 518060 P. R. China

## Abstract

The 3D hierarchical structure in catalysts not only the preserves intrinsic characteristics of each component, but also achieves increased specific surface area and active sites for the hydrogen evolution reaction (HER). Herein, we report a new strategy to synthesize efficient 3D hierarchical catalysts composed of Mo_2_C nanosheets and Co nanoparticles (H-Mo_2_C@Co). It was realized by using raw materials, defect-rich MoO_*x*_, Co(NO_3_)_2_·6H_2_O and 2-methylimidazole, to design Mo/Co bimetallic metal–organic frameworks (BMOFs), followed by pyrolysis at 800 °C. The defects in MoO_*x*_ induced preferential nucleation and growth of the BMOFs so that they can ensure the construction of a stable 3D hierarchical structure. Mo_2_C and Co have a synergistic effect in improving the HER *via* providing large surface areas (351.5 m^2^ g^−1^), more active sites and optimizing charge transfer. It can achieve 10 mA cm^−2^ at low overpotential over a wide pH range (144 mV in 0.5 M H_2_SO_4_ and 103 mV in 1.0 M KOH) and the properties can be well maintained in both acid and alkaline electrolyte after 2000 cycles. The hierarchical catalyst contains no noble metal, can be synthesized on a large scale and recycled by magnetic stirring, demonstrating great potential in water splitting, wastewater treatment, dye adsorption and other fields.

## Abstract 2

In this work, we report a new strategy to synthesize efficient 3D hierarchical catalysts composed by Mo_2_C nanosheets and Co nanoparticles (H-Mo_2_C@Co). The Mo_2_C and Co makes a synergistic effect in improving HER *via* providing large surface areas.

## Graphical abstract

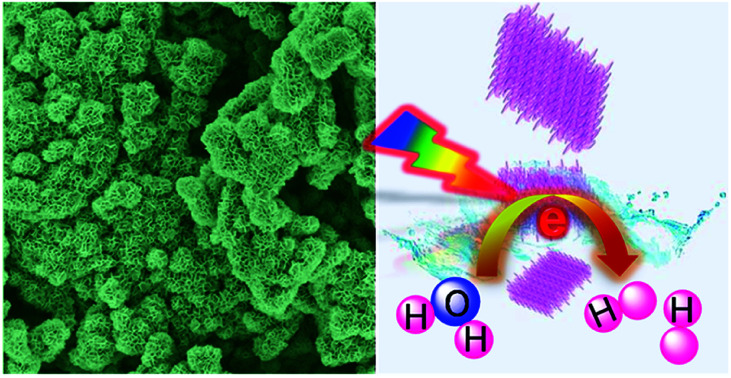

## 1. Introduction

With increasingly prominent environmental problems caused by traditional fossil and coal resources, it is imperative to look for green and sustainable energy sources.^1^ As an ideal energy carrier, hydrogen has the advantages of environmental friendliness and high energy density.^2,3^ It can be used as a clean fuel, as the final product is water, generating no pollutants. Moreover, it has important applications in the chemical, petroleum and metallurgical industries, such as industrial ammonia synthesis and hydrodesulfurization of petroleum.^3,4^ Therefore, the search for an efficient and energy-saving preparation method of hydrogen has become one of the research hotspots.^5^ Electrochemical water splitting is an amenable method to prepare pure hydrogen. Its suitability for both seawater and freshwater can reduce the cost to some extent. The preparation conditions are mild without further separation and purification, favoring cost-effective and large scale production. Therefore, preparing hydrogen from water will become one of the core technologies in the future hydrogen industry, which will have high social and economic benefits.

The key factor that influences the efficiency of water splitting is catalysts. Pt-based catalysts are considered as one of the most effective catalysts for the hydrogen evolution reaction (HER), but their exorbitant price, low earth abundance and unsatisfactory durability hinder their widespread utilization.^6^ Therefore, it is significant to investigate noble-metal-free alternatives with satisfactory performance in media with different pH values. Theoretical calculations show that cobalt (Co) nanoparticles have low adsorption free energy for hydrogen atoms and thus exhibit promising catalytic activity in HER. The problem is that they are prone to corrosion, passivation and aggregation.^7^ It was reported that encapsulating the metal nanoparticles by carbon layers can provide a solution for improving the chemical durability.^8^ They protects the internal nanoparticles from electrolytes, avoiding corrosion and oxidization from the external environment, and also prevent agglomeration with neighboring nanoparticles. However, the outermost carbon layer in this structure, is less modified and does not provide sufficient active sites, resulting in unsatisfactory catalytic activities.^9^ The recently reported molybdenum carbide (Mo_2_C) has better catalytic performance and exhibits excellent stability, corrosion resistance and high mechanical strength. If the protective carbon layer is converted to Mo_2_C nanocrystals, there will be more catalytic active sites. And the combination of Mo_2_C nanocrystals and Co nanoparticles at the molecular level will make a synergistic contribution, as the strong Mo–H bond will favor the adsorption of H^+^ ions, while the weak Co–H bond will improve conductivity and electron transfer. Therefore, Pt-like catalytic behaviors will be expected in this hybrid.

In addition to the chemical compositions, the catalytic properties also strongly depend on the structures of the electrocatalyst. Hierarchical structured catalysts are promising for hydrogen production, as it provides large surface area and abundant subunits, giving rise to rich surface active sites and high diffusion efficiency.^10^ Recently, several complex Mo_2_C hierarchical architectures have been synthesized by carbonizing Mo-dopamine precursors.^11^ They exhibit excellent electrocatalytic performance for HER with small overpotential in both acidic and basic conditions, as well as remarkable stability. To make hierarchical structure, metal–organic frameworks (MOFs) have been employed as effective sacrificial templates or scaffold.^12^ For instance, MoC_*x*_ nanooctahedrons were recently prepared from Cu-MOFs containing molybdenum-based polyoxometalates.^13^ Hu *et al.* presented an MOF template-engaged synthesis of CoS-nanosheet-assembled single-shelled nanoboxes (CoS-NS SSNBs) through two-ion exchange reactions.^14^ Li's group synthesized a novel HER electrocatalyst based on CoP nanoparticles embedded in a N-doped carbon nanotube hollow polyhedron (CoP/NCNHP).^15^ Inspired by these and theory of defect-driven preferential nucleation,^16^ we hypothesized that Mo-based nanosheets containing structural defects (*e.g.* oxygen vacancies) will provide opportunities for selective nucleation of Co atoms and form a bimetallic organic framework (BMOF), which provides a new way to synthesize functional composites.

In this work, we provide a new route to fabricate a novel 3D structure comprising hierarchical Mo_2_C nanocrystals and Co nanoparticles (H-Mo_2_C@Co). It was prepared by using MoO_*x*_ nanosheets with oxygen vacancy defects and Co(NO_3_)_2_·6H_2_O as Mo source and Co source to form Mo/Co BMOFs (MoCo-BMOFs) precursors followed by carburization. The resulting H-Mo_2_C@Co possesses abundant subunits, providing plenty of active sites on the exposed surfaces. The Mo_2_C in the present H-Mo_2_C@Co composite has large specific surface area (351.5 m^2^ g^−1^). Additionally, the hierarchical architecture facilitates the charge/mass transport in the materials during electrochemical processes. As a result, the hybrid exhibits outstanding catalytic performance with small overpotentials in both acidic electrolyte (*η*_10_ = 144 mV) and alkaline electrolyte (*η*_10_ = 103 mV), and great long-term cycling stability (negligible changes after 2000 cycles), making it become one of the most active transition-metal-based electrocatalysts for HER. Furthermore, benefiting from the 3D hierarchical structure, the low cost of the precursors, and the green route, the obtained H-Mo_2_C@Co offer very attractive prospects in HER and could be extended to the applications as adsorbents to remove pollutants from large volumes of acidic or alkaline solutions.

## 2. Results and discussion

**Fig. 1:**
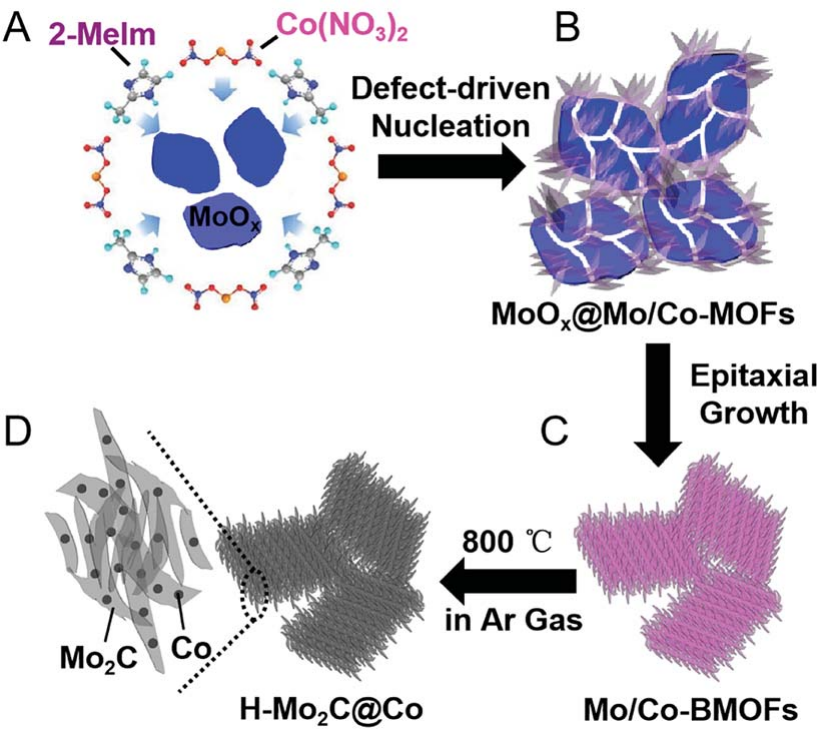
Schematic illustration of the fabrication processes of H-Mo_2_C@Co hybrid nanostructure. (A) Mixture solution of MoO_*x*_ nanosheets, 2-Meim and Co(NO_3_)_2_. (B) Partial transformation of MoO_*x*_ into MoCo-BMOFs, forming the intermediate product of MoO_*x*_@MoCo-BMOFs. (C) Prolonged reaction time allowing for continuous formation of MoCo-BMOFs *via* epitaxial growth. (D) Formation of H-Mo_2_C@Co hybrid structures by annealing at 800 °C in Ar gas.

A schematic illustration of the processes for fabricating H-Mo_2_C@Co hybrid nanostructures is shown in Fig. 1. First, the MoO_*x*_ nanosheets with oxygen vacancies were prepared as the Mo source by a method recently reported by a literature with slight modification.^17^ Then they were added into the aqueous solution of Co(NO_3_)_2_·6H_2_O and 2-methylimidazole (2-Meim) (Fig. 1A). The MoO_*x*_ has high surface energy, enabling preferential growth of MoCo-BMOFs precursor, which together with the rest of unreacted MoO_*x*_ forms an intermediate MoO_*x*_@MoCo-BMOFs (Fig. 1B). It is noted that the nucleation of MoCo-BMOFs nanosheets on the MoO_*x*_ surface is initiated at the oxygen defects of the MoO_*x*_ nanosheets. These sections feature a higher surface energy therefore enabling a preferential growth. With enough time, almost all the MoO_*x*_ turned into MoCo-BMOFs *via* epitaxial growth (Fig. 1C). Finally pyrolysis at 800 °C, the MoCo-BMOFs was converted to hierarchical structure of Mo_2_C nanocrystals and Co nanoparticles (H-Mo_2_C@Co) on carbon matrix (Fig. 1D). Unless specifically stated, the samples of H-Mo_2_C@Co mentioned below are prepared by these conditions.

To investigate the growth mechanism and the role of MoO_*x*_ in the formation of H-Mo_2_C@Co, several contrast experiments were conducted. As a comparison, we used MoO_3_ prepared by annealing MoO_*x*_ at 300 °C in air as Mo source and repeated the experiments. As illustrated in Fig. S1†, the MoO_3_ cannot react with 2-Meim, instead being covered by Co-MOFs (MoO_3_@Co-MOFs). This intermediate can only leads to the formation of porous Mo_2_C@Co (P-Mo_2_C@Co), whose structure is totally different from H-Mo_2_C@Co. These results indicate that the oxygen vacancies in MoO_*x*_ drives the reaction with 2-Meim and determine the formation of Mo/Co BMOF and the subsequent H-Mo_2_C@Co. During reaction, the oxygen vacancies were complemented by the N atoms in 2-Meim. The N atoms have strong coordination with Mo, so they can take Mo away from MoO_*x*_ and initiate the reaction. The oxygen vacancies of MoO_*x*_ were verified by the color contrast of the samples. Although the scanning electron microscopy (SEM) images of MoO_*x*_ and MoO_3_ (Fig. S2A and B†) show no big difference, the photographs of the powders (Fig. S2C and D†) exhibit obvious color contrast. The dark blue color of MoO_*x*_ power indicates the existence of rich oxygen vacancies, while grayish white MoO_3_ verifies their disappearance. Similar conclusions have been well documented in WO_2.83_ nanorods^18^ and TiO_2−*x*_ nanocrystals,^19^*i.e.*, the blue color of the transition metal oxide is derived from the characteristic external d-shell electrons caused by the oxygen vacancies. To further prove the existence of oxygen vacancies in MoO_*x*_ nanosheets, electron paramagnetic resonance (EPR) was measured on MoO_*x*_ nanosheets and MoO_3_ nanosheets at room temperature under different magnetic fields (Fig. S3†). It shows that MoO_*x*_ nanosheets, different from MoO_3_ powder, have significant EPR signal at *g* = 1.998, due to magnetic dipoles. It is assigned to unpaired electrons on the oxygen vacancies, which is also reported by other work.^20^ The signal at a *g* = 1.935 is also detected and attributed to Mo^5+^.^21^ In addition, it is worth mentioning that the involvement of Co ions at the first step is also important. Without Co ions, the MoO_*x*_ would turn into Mo infinite coordination polymer particles (Mo-ICPs) (Fig. S4A†). The XRD pattern of Mo-ICPs shows no typical peaks, indicating it is amorphous (Fig. S4B†). After pyrolysis, the ICPs collapses and is replaced by highly agglomerated Mo_2_C nanocrystals (Fig. S4C†). Therefore, the addition of Co helps stabilize the 3D hierarchical structure.

**Fig. 2:**
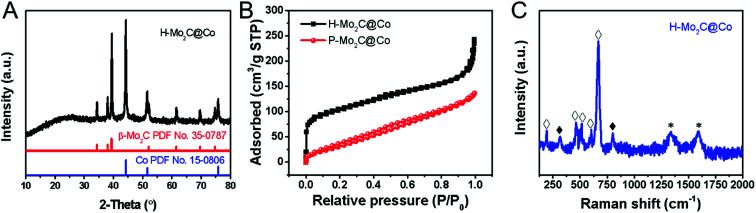
(A) Powder XRD patterns of MoO_*x*_ nanosheets, Co-MOFs, MoCo-BMOFs and H-Mo_2_C@Co hybrid. (B) N_2_ adsorption–desorption isotherm curve of H-Mo_2_C@Co and P-Mo_2_C@Co. (C) Raman spectra of the H-Mo_2_C@Co hybrid. Black and hollow squares denote the peaks from Mo_2_C and Co, respectively.

The phase transformations of the samples were confirmed by XRD measurements (Fig. 2A and S5†). As for MoO_*x*_ nanosheets, it is assigned to the orthorhombic phase (JCPDS No. 05-0508), which is crystallized in a layered structure composed of MoO_6_ octahedra. The XRD peaks of Co-MOFs and MoCo-BMOFs were not substantially different because the product of MoO_*x*_ reacting with 2-Meim was amorphous Mo-ICPs. After annealing, the MoCo-BMOFs were fully transformed into the Mo_2_C nanocrystals and Co nanoparticles, as confirmed by the XRD patterns (Fig. 2A) with according diffraction peaks at 34.4°, 38.0°, 39.4°, 52.1°, 61.5° and 69.6° (110) for β-Mo_2_C (PDF No. 35-0787) and peaks marked with # for metallic Co (PDF No. 15-0806). No peaks from impurity phases can be detected, demonstrating high purity of hierarchical Mo_2_C@Co hybrid. The peak located at 25.8° is assigned to the formation of partially carbon derived from the organic precursor, because the peak is weak and wide, indicating that the degree of graphitization is not high. The XRD of P-Mo_2_C@Co (Fig. S6†) is similar to that of H-Mo_2_C@Co, indicating their similarity in components. However, their active surface area is totally different. The Brunauer–Emmett–Teller specific surface (*S*_BET_) derived from N_2_ adsorption–desorption isotherm curves is shown in Fig. 2B. The H-Mo_2_C@Co hybrids show *S*_BET_ of 351.5 m^2^ g^−1^, which is much larger than that of P-Mo_2_C@Co hybrid nanosheets (130.2 m^2^ g^−1^). To our knowledge, this is one of the largest specific surface areas of the synthesized Mo_2_C materials reported so far.^22^ The large *S*_BET_ of H-Mo_2_C@Co hybrids is favorable in transfer of the reactants and enhancing HER performance. Its Raman spectrum in Fig. 2C displays strong characteristic bands of Co metal at 192.3, 467.3, 515, 601.6 and 668.5 cm^−1^ (hollow square),^8*c*,23^ and weak peaks of Mo_2_C at ∼300 and ∼800 cm^−1^ (black square).^24^ The broad peaks at 1343.4 and 1591.3 cm^−1^ are attributed to D and G bands of carbon. The D band is attributed to the formation of defects and lattice distortion while the G band is associated with the in-plane vibration of sp^2^ carbon atoms, thus the intensity ratio of *I*_D_/*I*_G_ reflects the degree of in-plane defect concentration or edge defect of carbon.^25^ These defects are generally the center of catalytic activity. For carbon materials, pyridinic-N and pyrrolic-N are the sites for catalytic activity.^26^ The existence of pyridinic-N and pyrrolic-N will be further verified in the XPS below.

**Fig. 3:**
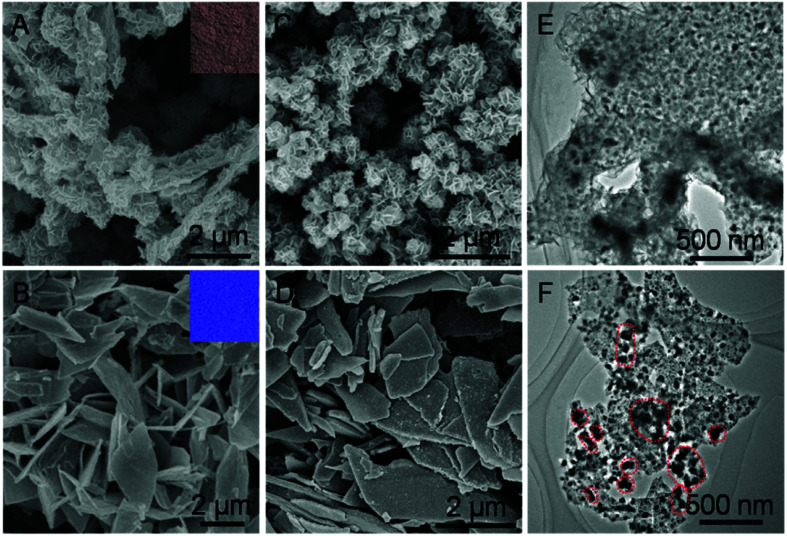
(A and B) SEM images of the MoCo-BMOFs precursor and MoO_3_@Co-MOFs precursor (inset: photograph of powder). (C and D) SEM images and (E and F) TEM images of H-Mo_2_C@Co hybrid and P-Mo_2_C@Co hybrid, respectively. Dark spots are typical Co particles.

Taking H-Mo_2_C@Co as a model sample, we further conducted scanning electron microscopy (SEM) and transition electronic microscopy (TEM) to investigate the structure of Mo_2_C@Co hybrids. As shown in Fig. 3A and B, employing MoO_*x*_ and MoO_3_ nanosheets as Mo sources, generate brown MoCo-BMOFs and blue MoO_3_@Co-MOFs precursors, respectively. The morphology of the two precursors is completely different, mainly due to the difference of the number of oxygen defects in MoO_*x*_ and MoO_3_. Specifically, the MoCo-BMOFs precursor features in tremella-like loose structure consisting of ultrathin nanosheets with a thickness <20 nm, as shown in Fig. 3A. In contrast, the MoO_3_@Co-MOFs have plate-like tight shapes (Fig. 3B) with subunits as core–shell structures (Fig. S7†). The structure of the precursors determines the basic framework of the final products so as to influence their HER performance. Inherited from the basic structure of the precursor, the H-Mo_2_C@Co begins to possess more dispersed flower-like particles (Fig. 3C), while P-Mo_2_C@Co becomes a little rougher due to pores (Fig. 3D). The TEM image shows that the H-Mo_2_C@Co was wrapped around by ultrathin Mo_2_C nanosheets and each sheet is uniformly decorated with Co nanoparticles with a size of ∼20 nm (Fig. 3E). To the contrary, the Co particles (dark spots) in P-Mo_2_C@Co have various sizes and are non-uniformly distributed on the nanoplates (Fig. 3F). The H-Mo_2_C@Co is able to take full advantages of the hierarchical structure with a higher specific surface area and a more uniform dispersion of the Co nanoparticles.

**Fig. 4:**
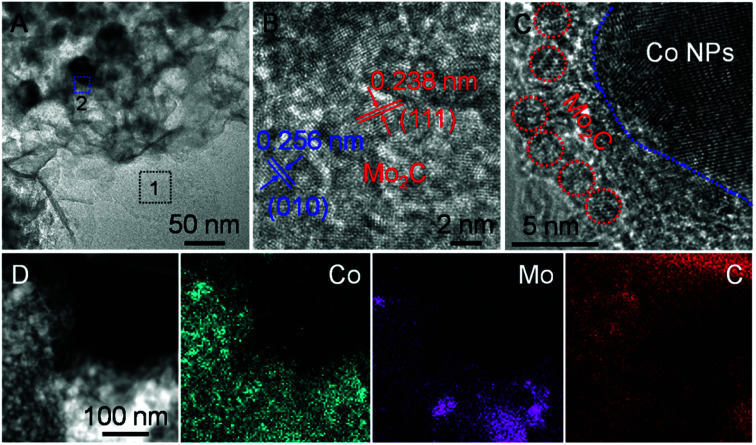
(A) Zoom-in TEM image of H-Mo_2_C@Co. (B and C) HRTEM images taken from the representative regions outlined by 1 and 2 in (A). (D) EDS elemental mapping from STEM of H-Mo_2_C@Co.

Zooming in the TEM image (Fig. 4A), it verifies that overall dispersion of Co nanoparticles on the Mo_2_C nanosheets are homogeneous for H-Mo_2_C@Co. The high-resolution TEM (HRTEM) image taken from Region 1 reveals the lattice fringes with d-spacing of 0.256 and 0.238 nm, which correspond to (010) and (111) plane of the crystalline Mo_2_C (Fig. 4B). The entire Mo_2_C ultrathin sheet has lattice fringes with different orientations, indicating that it is polycrystalline. It is noted that the aggregation of Co nanoparticles at few local positions is unavoidable, like Region 2, albeit that the hierarchical structure has minimized their aggregations. In these regions, it looks like Mo_2_C nanocrystals surrounding the Co nanoparticles, as this part of Mo_2_C nanocrystals is much smaller than Co nanoparticles (Fig. 4C). Energy dispersive X-ray spectroscopy (EDS) analysis (Fig. S8†) confirms the main composition of C, N, Co and Mo elements in the hybrids, further supporting the formation of Mo_2_C and Co. The corresponding elemental mapping (Fig. 4D) reveals the uniform distribution of Co, Mo and C, which verifies the even integration of Mo_2_C and Co nanoparticles in the carbon matrix. It can be seen that the close contact of metal Co and Mo_2_C will facilitate the electron transport between them. By contrast, in the P-Mo_2_C@Co hybrid, the Co nanoparticles are seriously agglomerated (Fig. S9†), and the carbon matrix is much thicker than the nanosheets, which may result in reduced electron transport between them.

**Fig. 5:**
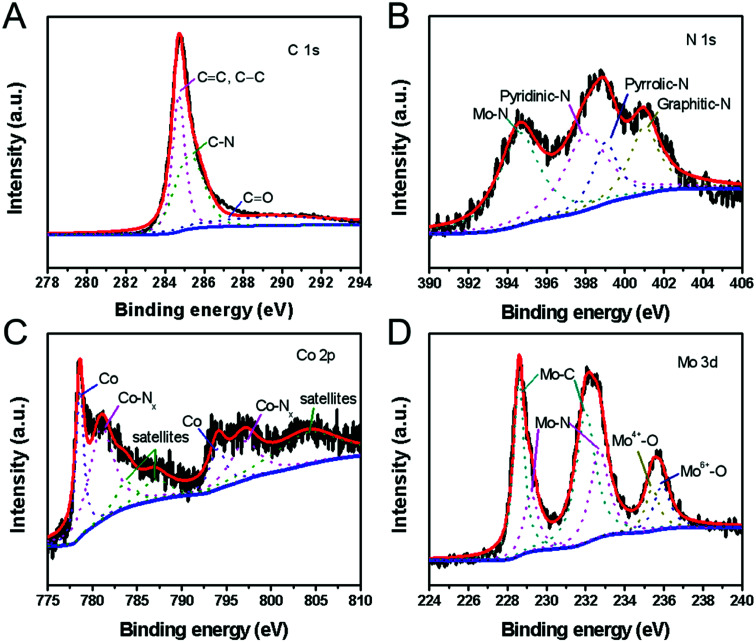
(A) C 1s XPS spectrum, (B) N 1s XPS spectrum, (C) Co 2p XPS spectrum, and (D) Mo 3d XPS spectrum of the H-Mo_2_C@Co hybrid. The red curve is the sum, the blue curve is the baseline, and the dotted curves are the fitted.

The molecular structure and element states of the H-Mo_2_C@Co hybrid were also studied by X-ray photoelectron spectroscopy (XPS). The high-resolution XPS spectrum of C 1s can be deconvoluted into three subpeaks with binding energy of 284.6, 285.3 and 289.8 eV, which are attributed to carbon in C=C or C–C, C–N, and C=O bonds, respectively (Fig. 5A).^27^ The N 1s spectrum (Fig. 5B) reveals the presence of four types of N species, *i.e.*, the pyridinic N (398.2 eV), the pyrrolic N (399.4 eV) and graphitic N (401.1 eV) in the H-Mo_2_C@Co hybrids,^27^ and N–Mo bond (394.6 eV).^28^ The Co 2p XPS spectrum is resolved into two pairs of 2p_3/2_/2p_1/2_ doublets for metallic Co (778.5/793.6 eV) and N-coordinated Co^2+^ (Co–N_*x*_) (781.0/796.3 eV) with an energy separation of 15.1 eV (Fig. 5C).^29^ In addition, the peaks with binding energies of 783.6 eV, 786.6 eV and 803.9 eV are caused by the satellite peaks of the Co^3+^ and Co^2+^ ions.^30^ The high-resolution Mo 3d spectrum (Fig. 5D) was deconvoluted into six peaks, corresponding to Mo^2+^ (228.6 and 232.0 eV), Mo^3+^ (229.2 and 232.7 eV) and Mo^*δ*+^ (235.4 and 235.9 eV) species. Mo^2+^ is from carbide of Mo, and Mo^3+^ is related to molybdenum nitrides, which are known to serve as active sites for HER.^31^ Mo^*δ*+^ can be assigned to oxides of Mo, *e.g.*, excessive MoO_*x*_ or the surface oxidation of Mo species. According to XPS analysis, Mo_2_C and the carbon matrix are slightly doped by N from 2-Meim. Particularly, some of the MoN and CoN formed between them can improve their catalytic performance through strong electron interaction with Mo_2_C.^32^ In addition, pyridinic N is the most active composition in electrochemical, and its content often determines the electrochemical performance of N-doped carbon.^33^ Therefore, the presence of N doping provides the possibility to improve the electrocatalytic performance.^34^

The MoCo-BMOFs are important intermediates to form H-Mo_2_C@Co hybrid. Their size and morphology can be readily tailored by controlling the reaction time (Fig. S10†). At the beginning of the reaction (*e.g.* 1 h), MoCo-BMOFs heterogeneously nucleated and grew at the interface of the MoO_*x*_ nanosheets (Fig. S10A†). The MoO_*x*_ nanosheets were still smooth without observable pores. When the reaction time was prolonged to 4 h, irregular MoCo-BMOFs nanosheets grew on the surface of MoO_*x*_ nanosheets and there were observable significant etching in the MoO_*x*_ templates (Fig. S10B†). By further increasing the reaction time, the hierarchical nanosheets of the MoCo-BMOFs became more and more obvious and perfect, and the size of the sheets gradually increased to ∼200 nm at 8 h (Fig. S10C†). However, there are still a small amount of MoO_*x*_ fragments. After 12 h reaction, the hierarchical MoCo-BMOFs nanosheets remained uniform while the MoO_*x*_ nanosheets were etched out (Fig. S10D†). Here, the sheet morphology is related to the intrinsic crystallographic structure of MoCo-BMOFs. The MoCo-BMOFs seeds from the interfacial reaction of surface defects, which are nucleation centers, allow the subsequent adsorption of Mo and Co and the crystal growth within the plane. The MoCo-BMOFs serve as scaffold to support 3D hierarchical structures, which help in stabilization and avoid collapsing.

**Fig. 6:**
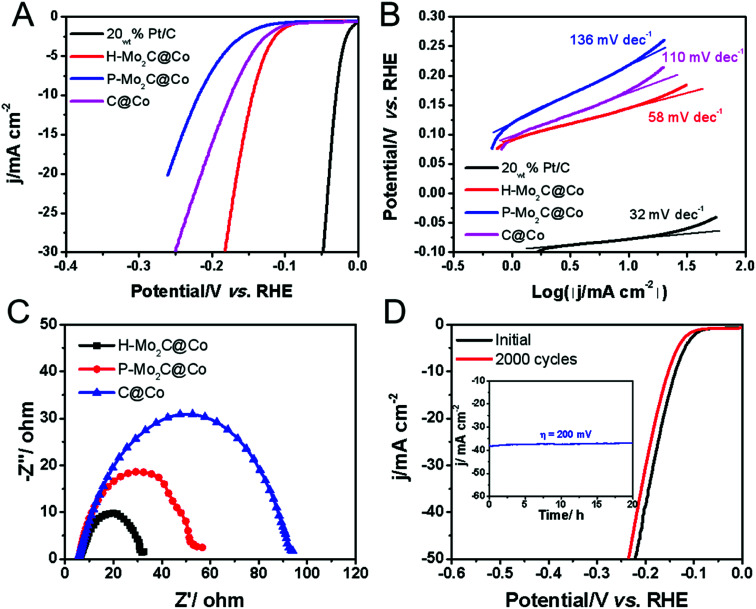
Polarization curves (A) of H-Mo_2_C/Co, P-Mo_2_C/Co, C@Co and 5% Pt/C at a scan rate of 5 mV s^−1^ in 0.5 M H_2_SO_4_ and the corresponding Tafel plots (B). (C) Nyquist plots (at *η* = 250 mV) of H-Mo_2_C@Co hybrids, P-Mo_2_C/Co and C@Co obtained by EIS. (D) Polarization curves of H-Mo_2_C@Co before and after 2000 potential cycles at a scan rate of 100 mV s^−1^. Inset: long-term durability tests at an overpotential of 200 mV for 20 h.

The electrocatalytic HER performances were evaluated in acidic electrolyte by using a graphite rod as the counter electrode and carbon fiber paper (CFP) as the conductive substrate due to its negligible HER activity. The HER polarization curves of H-Mo_2_C@Co, P-Mo_2_C@Co, C@Co derived from Co-MOFs and commercial Pt/C are obtained at a voltage sweeping rate of 5 mV s^−1^ in 0.5 M H_2_SO_4_ (Fig. 6A). The as-prepared MoO_*x*_ nanosheets exhibit poor HER activity, *i.e.*, potential is > −0.5 V as the reduction current reaches to 10 mA cm^−2^ (Fig. S11, see ESI†). However, the catalytic activity of H-Mo_2_C@Co is dramatically enhanced (Fig. 6A). It has a low overpotential (*η*_10_) of 144 mV at a current density of 10 mA cm^−2^ in 0.5 M H_2_SO_4_ solutions, which are superior to those from P-Mo_2_C@Co (*η*_10_ = 217 mV) and C@Co (*η*_10_ = 175 mV). Compared to commercial Pt/C catalyst (*η*_10_ = 29 mV), the H-Mo_2_C@Co still has much room to get improved. Accordingly, the Tafel plots representing the HER kinetics of the above catalysts displays the same sequence (Fig. 6B). They were plotted by using the equation *η* = *a* + *b* log(|*j*|), where *a* is the intercept and *b* is the Tafel slope. The H-Mo_2_C@Co has a Tafel slope of 58 mV dec^−1^, indicating it follows the Volmer–Heyrovsky catalytic mechanism.^35,36^ Although Tafel slope value is slightly larger than that of Pt/C catalyst (32 mV dec^−1^), it is reduced to ∼1/2 of P-Mo_2_C@Co (136 mV dec^−1^) and C@Co (110 mV dec^−1^), demonstrating improved HER kinetics in H-Mo_2_C@Co. The dramatic change in Tafel slope from H-Mo_2_C@Co to P-Mo_2_C@Co indicates a change in the mechanism. It can be observed from Fig. S12† that the Co nanoparticles in P-Mo_2_C@Co are significantly more agglomerated, while the Co nanoparticles in H-Mo_2_C@Co are well dispersed. The morphology of Co nanoparticles are different between the two materials, which may lead to different kinks/stepped site exposures, thus changing the binding energy of the protons to the surface and changing the mechanism of HER.

The double-layer capacitance (*C*_dl_), which is proportional to the electrochemically active surface area (ECSA),^11*d*,31^ was also measured to probe the advantages of the H-Mo_2_C@Co hybrid catalyst. Derived from the cyclic voltammograms (CVs) with varied scan rates (Fig. S13A–D†), H-Mo_2_C@Co hybrid presented a much higher *C*_dl_ of 9.08 mF cm^−2^ than those of the P-Mo_2_C@Co (2.84 mF cm^−2^) and C@Co (5.14 mF cm^−2^) (Fig. S13E†). The high *C*_dl_ value implies the existence of abundant active sites for HER on the hierarchical hybrid structure. Additionally, electrochemical impedance spectroscopy (EIS) measurements demonstrate that the H-Mo_2_C@Co hybrid has the lowest charge transfer resistance (*R*_ct_) of 26.4 Ω, indicated the better HER kinetics and expedited faradaic process. As for the P-Mo_2_C@Co and C@Co, the values were 53 and 95 Ω, respectively (Fig. 6C). As the overpotential increases, the *R*_ct_ becomes smaller and smaller, which also indicates that the electron transfer is accelerated and the hydrogen production rate is gradually increased (Fig. S13F†). The durability is another important criterion for a HER catalyst. The long-term stability of H-Mo_2_C@Co was revealed by continuous cyclic voltammetry performed at 0.05 to −0.50 V in 0.5 M H_2_SO_4_ (Fig. 6D). The polarization curve only shows a very slight negative shift after the 2000 CVs cycles. Additionally, the current density exhibits negligible change after 20 h testing (inset in Fig. 6D). Meanwhile, the chronopotentiometric (CP) curve recorded at a constant cathodic current density of −10 mA cm^−2^ over a course of 20 h shows a slight increase of the overpotential, also demonstrating the excellent catalytic durability of the hybrid catalyst electrode (Fig. S14†). This exceptional durability promises for practical applications of the catalyst in the water-splitting field. The FESEM and TEM images of H-Mo_2_C@Co after the stability test indicates that the hierarchical structure was retained, but the sharp edges of the nanosheets became roundish (Fig. S15A and B†) due to the surface changing of Mo species to amorphous oxide during the HER tests. The HER cycle test did not change the valence states of Co, indicating that Co nanoparticles had high stability in the cycle tests. However, after long-term testing, the Mo species was not very stable (Fig. S15C–F†). Solving the problem that Mo easily oxidizes is still a big challenge.^37^

Before the above research, we had made a series of H-Mo_2_C@Co by tuning the dosages of MoO_*x*_ (200, 300 and 400 mg) and annealing temperatures of precursors (700, 800 and 900 °C). We had found that the two factors play an important role in the HER performances. XRD patterns and N_2_ adsorption–desorption isotherm curves of these samples are shown in Fig. S16 and S17†. Typical diffraction peaks of Mo_2_C@Co are all observed in these samples, suggesting the accomplishment of solid-state reactions under these fabrication conditions (Fig. S16†). The H-Mo_2_C@Co prepared by using 300 mg MoO_*x*_ and precursor annealed at 800 °C displayed the largest surface area of 351.5 m^2^ g^−1^ (Fig. S17†), and the optimal performance in HER (Fig. S18†). These studies suggest that a balance between the amounts of MoO_*x*_ nanosheet and annealing temperature is needed for preparing an excellent HER electrocatalyst.

In order to expand the application range of the catalysts, we also explored their HER performance in alkaline electrolyte (1.0 M KOH) and their magnetism. The performance of H-Mo_2_C@Co in alkaline electrolyte is more or less the same as in acid electrolyte (Fig. 6). It shows overpotential of 103 mV at a current density of 10 mA cm^−2^ and the Tafel slope of 87 mV dec^−1^ (Fig. S19†). These values are slight larger than those of commercial Pt/C, but much smaller than those of P-Mo_2_C@Co, MoO_*x*_ nanosheets and C@Co. The durability of H-Mo_2_C@Co in alkaline electrolyte is also excellent and the performance has no degradation after 2000 cycles. According to the comparison between molybdenum carbide-based catalysts toward water splitting, we can conclude that H-Mo_2_C@Co hybrid is an excellent functional electrocatalyst. Its performance in both acidic and alkaline electrolytes is superior to most previously reported MoCo-based electrocatalysts for HER.^38–41^

In addition to electrochemical properties, magnetism is also tested. Fig. S20A† displays the room-temperature magnetic hysteresis loops of H-Mo_2_C@Co and P-Mo_2_C@Co. The saturation magnetization (*M*_s_), remanent magnetization (*M*_r_) and coercivity (*H*_c_) values for H-Mo_2_C@Co (P-Mo_2_C@Co) are approximately 68.8 (27.7) emu g^−1^, 15 (5.8) emu g^−1^ and 0.34 (0.34) kOe, respectively, indicating the hierarchical hybrid has better ferromagnetism. Based on the superior magnetic properties and high specific surface area (351.5 m^2^ g^−1^, Fig. 2B), the H-Mo_2_C@Co hybrids may have great potential as an efficient adsorbent in wastewater treatment. Fig. S20B† verifies that the H-Mo_2_C@Co has a strong adsorption effect on arsenic (As) ions in water. In addition, the H-Mo_2_C@Co hybrids can be conveniently separated from the solution by simple use of an external magnet, and do not need filtration and centrifugation. This indicates that the hybrid can be easily recycled, which is important for practical water treatment.

## 3. Conclusion

In summary, we report a new strategy to synthesize 3D hierarchical architecture consisting of Mo_2_C nanocrystals and Co nanoparticles and obtained an excellent electrocatalytic performance in HER. MoCo-BMOFs precursors were first synthesized *via* selective nucleation at oxygen vacancies on the MoO_*x*_ surface. The H-Mo_2_C@Co was obtained by directly carburizing the MoCo-BMOFs precursors under an Ar atmosphere. The hierarchical hybrid catalyst shows excellent catalytic activity and long-term durability (>2000 cycles) for the HER in both acidic (*η*_10_ = 144 mV) and alkaline (*η*_10_ = 103 mV) electrolyte. The synergistic effect between Mo_2_C and Co nanoparticles, their ultrahigh specific surface area (351.5 m^2^ g^−1^) and their strong chemical coupling with highly conductive carbons are considered to contribute the excellent HER. In addition, the as-prepared H-Mo_2_C@Co hybrid can be rapidly collected using an external magnet due to its ferromagnetic property and can be recycled. This work will open up new opportunities to couple cheap species for the creation of high-performance electrocatalysts, which is greatly needed for sustainable development.

## 4. Experimental section

### 4.1 Synthesis of MoO_*x*_ nanosheets

The MoO_*x*_ nanosheets was prepared by a method reported by a literature with slight modification.^17^ In brief, 6 mmol of molybdenum metal powder was added to a Teflon vessel (100 mL) containing 70 mL of ethanol. Then 10 mL of H_2_O_2_ was introduced and magnetically stirred for about 15 min to form the transparent yellow solution. The Teflon vessel was then sealed in stainless steel autoclave, heated and maintained at 160 °C for 12 h. After cooling down to R.T. naturally, the product was collected by centrifugation, rinsed with ethanol for three times and finally dried at 60 °C overnight. The MoO_3_ nanosheets were obtained by heat-treating oxygen-defective MoO_*x*_ nanosheets at 300 °C for 3 h in air.

### 4.2 Synthesis of MoCo-BMOFs and MoO_3_@Co-MOFs precursors

The MoCo-BMOFs precursor was synthesized at R.T. First of all, 200, 300 or 400 mg of the as-prepared MoO_*x*_ nanosheets was added into the aqueous solution of Co(NO_3_)_2_·6H_2_O (40 mL, 582 mg). Then another aqueous solution containing 2-methylimidazole (40 mL, 1.312 g) was quickly added into the above suspension. After reaction for 1, 4, 8 or 12 h, the sample was collected by centrifugation and washed with water and ethanol for several times before drying at 60 °C overnight. The core–shell MoO_3_@Co-MOFs precursor was synthesized with the same procedure as MoCo-BMOFs precursor except for the use of MoO_3_ nanosheets as Mo source.

### 4.3 Synthesis of H-Mo_2_C@Co and P-Mo_2_C@Co hybrid nanostructures

High temperature carburizing process was conducted. To obtain H-Mo_2_C@Co, MoCo-BMOFs precursor was annealed at 700, 800 or 900 °C under Ar (100 sccm) gas flow for 3.5 h with a ramp rate of 2 °C min^−1^. To obtain P-Mo_2_C@Co, MoO_3_@Co-MOFs precursor was annealed at 800 °C through a similar process. After preparation, all the samples were treated with dilute H_2_SO_4_ for 4 hours at R.T., in order to ensure a stable sample composition during characterization.

### 4.4 Characterization

The morphology, structure and composition of the samples were characterized by field emission SEM (FESEM, ZEISS-Merlin SEM), transmission electron microscopy (TEM), energy dispersive X-ray spectroscopy (EDS) (FEI Tecnai G2 F20 Super-Twin TEM), X-ray diffraction (XRD, Bruker D8 Discover diffractometer, using Cu Kα radiation, *λ* = 1.540598 Å), X-ray photoelectron spectroscopy (XPS, ESCALAB 250Xi-resolved XPS system (Al Kα, *hν* = 1486.6 eV)), and Raman scattering (WITEC-Alpha 300 R) with excitation of 532 nm laser. The N_2_ adsorption–desorption isotherm was conducted by using NOVOE 4000. Inductively coupled plasma-atomic emission spectroscopy (ICP-AES) was performed on an Agilent Technologies 7700 series instrument. The electron paramagnetic resonance (EPR) spectrum was recorded on a JEOL JES-FA200 EPR spectrometer (300 K, 9064 MHz, 0.998 mW, X-band).

### 4.5 Electrocatalytic study

Electrochemical measurements for HER were carried out in a three-electrode configuration with CHI 760E electrochemical workstation. The catalyst electrodes were prepared by dropping catalyst ink onto a carbon fiber paper (CFP). Graphite rod and saturated calomel electrode (SCE) were used as the counter and reference electrodes, respectively. Under alkaline conditions, the reference electrode is Hg/HgO. The catalyst ink was prepared by mixing 5 mg catalysts with 760 μL water, 200 μL ethanol and 40 μL Nafion solution (5 wt%) under sonication. Then, 50 μL catalyst ink was transferred onto the CFP substrate with a catalyst loading of ∼1.25 mg cm^−2^. A similar method was used to prepare a commercial Pt/C (20 wt%, Johnson Matthey) catalyst for comparison. After drying, the loading amount was about 1.25 mg cm^−2^. Before the electrochemical tests, the electrolyte (0.5 M H_2_SO_4_ or 1.0 M KOH) was bubbled with nitrogen gas for 30 min. The polarization curves were obtained from the linear sweep voltammetry with a scan rate of 5 mV s^−1^. It should be noted that we defined the potential at a current density of 1 mA cm^−2^ as the onset potential. All the data were *iR*-corrected unless it is specifically stated.

#### *iR* correction

To correct the ohmic drop, all measured potentials were calibrated by using equation *E*_cor_ = *E* − *iR*, where *E*_cor_ is corrected potential, *E* is measured potential, *i* is current and *R* was the contact resistance derived from EIS data.

## Conflicts of interest

There are no conflicts to declare.

## Supplementary Material

RA-010-D0RA01197E-s001
