# Supplementary material for: Defect-induced nucleation and epitaxial growth of a MOF-derived hierarchical Mo2C@Co architecture for an efficient hydrogen evolution reaction
Source: RSC Adv. 2020 Apr 6;10(23):13838–47. doi: 10.1039/d0ra01197e (PMC9051579; doi:10.1039/d0ra01197e)
Supplement: RA-010-D0RA01197E-s001 [file RA-010-D0RA01197E-s001.pdf]

Electronic Supplementary Material (ESI) for RSC Advances.
This journal is © The Royal Society of Chemistry 2020

Electronic Supporting Information

Defect-Induced Nucleation and Epitaxial Growth of MOF-Derived Hierarchical Mo_2C@Co Architecture for Efficient Hydrogen Evolution Reaction

Linfei Zhang[a, b], Jingting Zhu,[a] Yumeng Shi,[a] Zhuo Wang[a,] and Wenjing Zhang[a,] **

a. Institute of Microscale Optoelectronics, Shenzhen University, Shenzhen 518060, P. R. China

b. College of Physics and optoelectronic Engineering, Shenzhen University, Shenzhen 518060, P. R. China

* Address correspondence to wjzhang@szu.edu.cn and wzhuo@szu.edu.cn

[#] The authors made equal contribution.

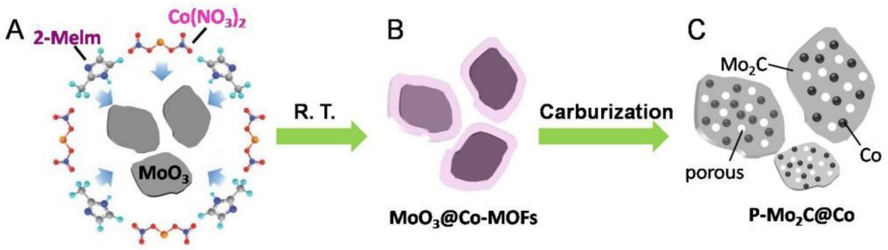

Figure S1. Schematic illustration of the synthetic processes of P-Mo_2C@Co hybrid nanostructure. The MoO_3 nanosheets leads to the formation of core-shell MoO_3@Co-MOFs precursor and the final product of porous Mo_2C@Co.

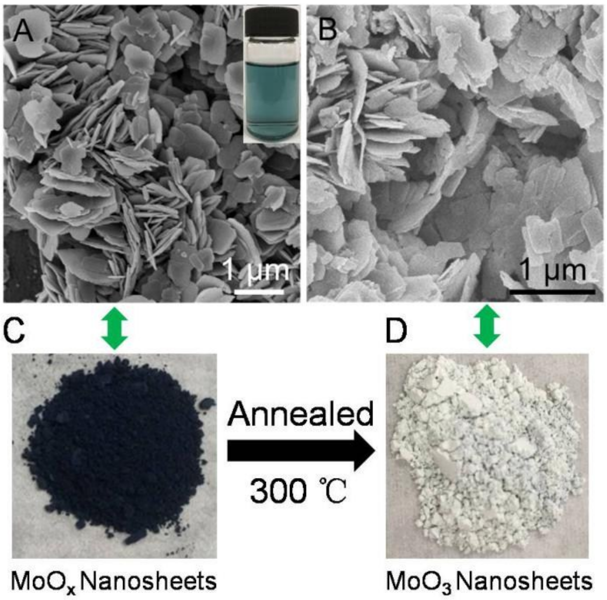

Figure S2. SEM images of the MoO_x nanosheets (A) and MoO_3 nanosheets (B), and corresponding to the photograph of powder (C and D). The inset in (A) shows a photograph of the MoO_x product dispersed in ethanol.

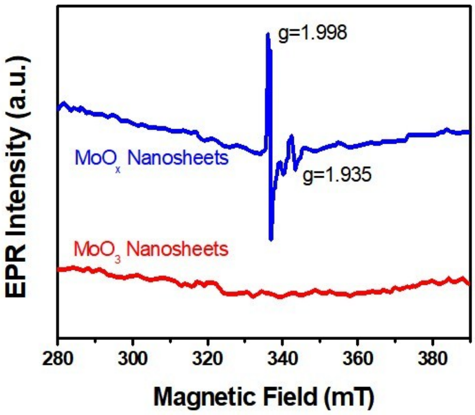

Figure S3. X-band EPR spectrum of MoO_x and MoO_3 nanosheets recorded at room temperature.

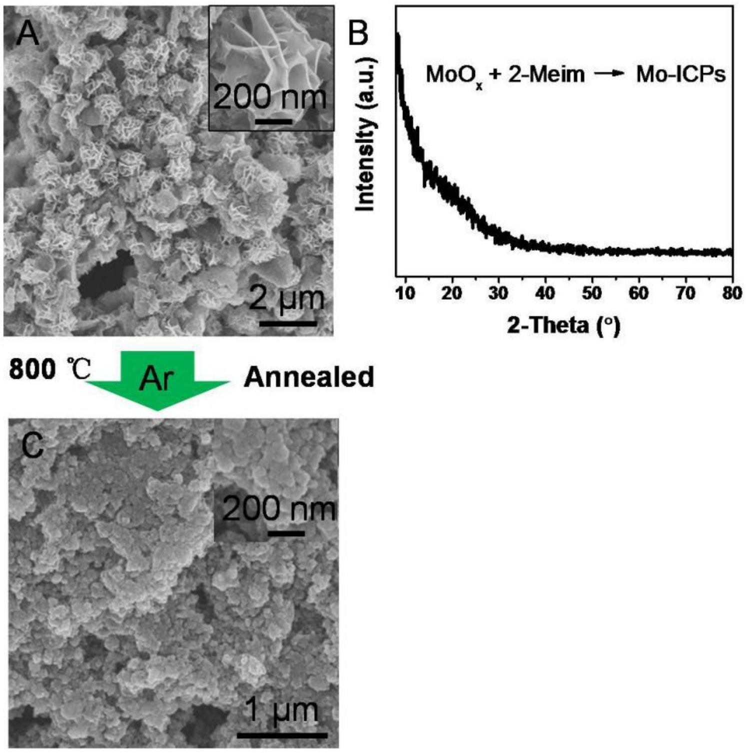

Figure S4. SEM image (A) and XRD pattern (B) of the Mo-ICPs. The powder X-ray diffraction data for the Mo-ICPs indicates that it is amorphous and not crystalline, which is the difference between ICPs and MOFs. (C) Pyrolysis at 800 ^{o}C, the Mo-ICPs was converted to highly agglomerated Mo_2C NCs. The inset of (A) shows the high-magnification SEM images.

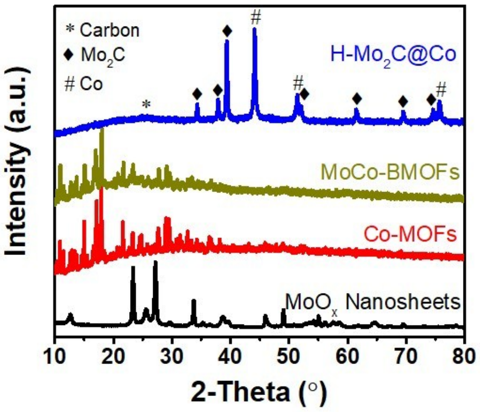

Figure S5. Powder XRD patterns of MoO_x nanosheets, Co-MOFs, MoCo-BMOFs and H-Mo_2C@Co hybrid.

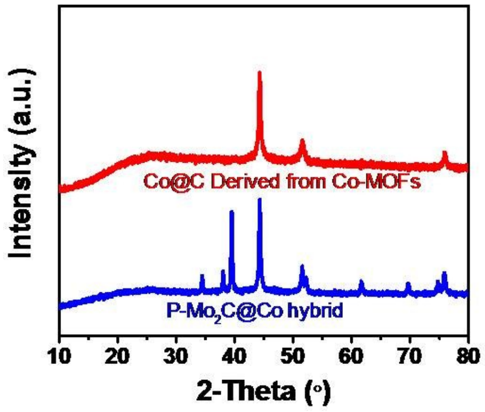

Figure S6. XRD pattern of the P-Mo_2C@Co hybrid and Co@C derived from Co-MOFs.

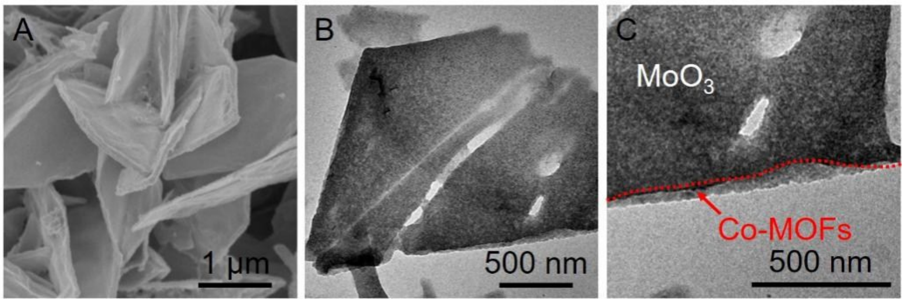

Figure S7. SEM image (A) and TEM images (B, C) of the MoO_3@Co-MOFs

precursor. The figures show that the Co-MOFs layer could be coated on surface of the MoO_3 nanosheets.

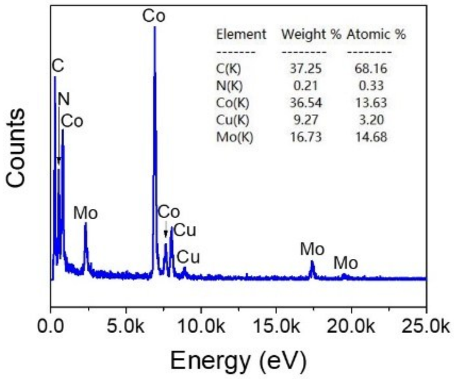

Figure S8. EDS spectrum of the H-Mo_2C@Co. The inset is element percentage.

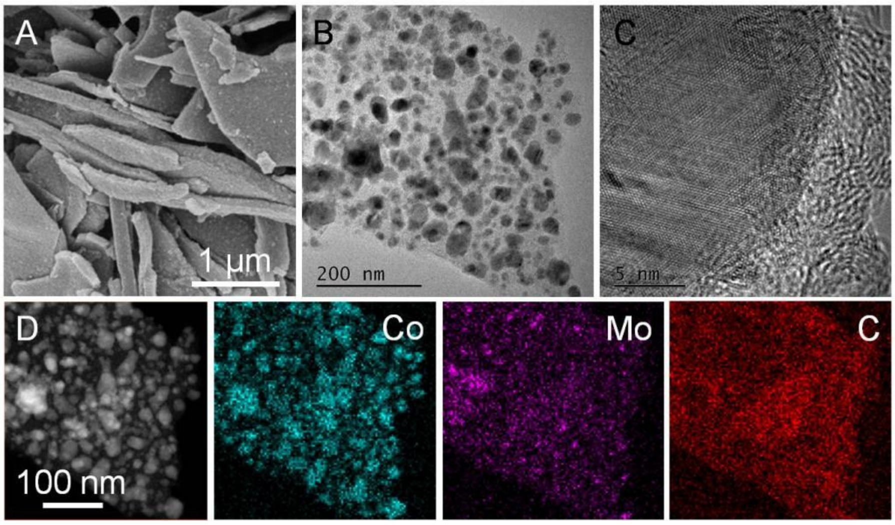

Figure S9. (A) SEM image, (B) TEM images, and (C) HRTEM image, (D) EDS mapping of the P-Mo_2C@Co and each element.

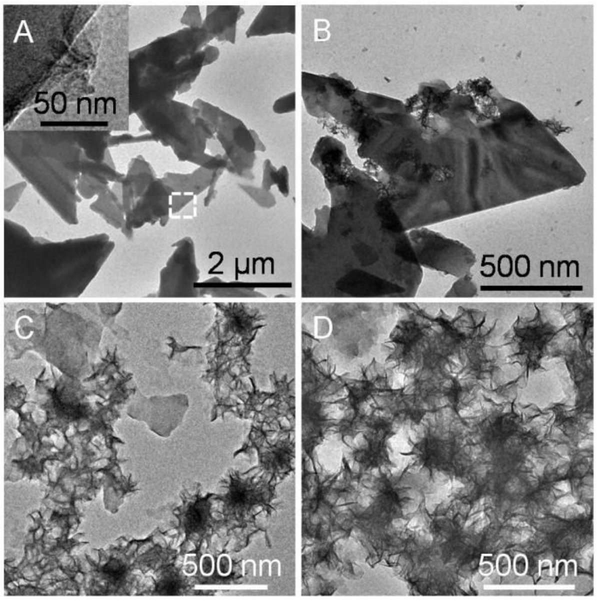

Figure S10. Typical TEM images of MoCo-BMOFs products after reaction for (A) 1 h (inset: magnified image from white frame), (B) 4 h, (C) 8 h and (D) 12 h.

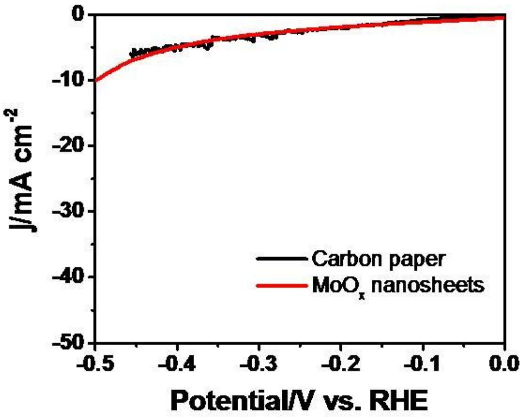

Figure S11. Polarization curve of bare carbon paper and MoO_x nanosheets in 0.5 M H_2SO_4 for evaluating HER activity.

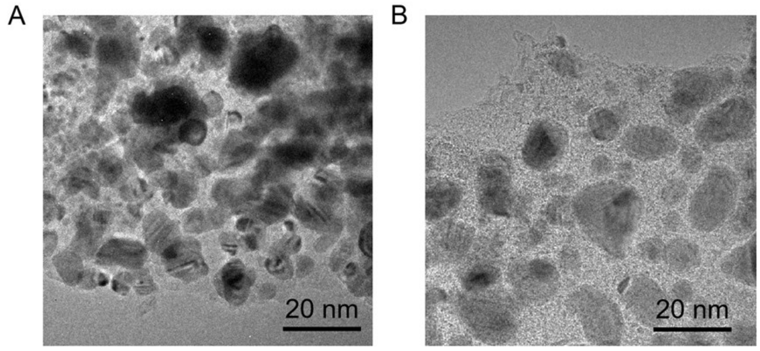

Figure S12. (A) and (B) are high-magnification TEM images of P-Mo_2C@Co and H-Mo_2C@Co.

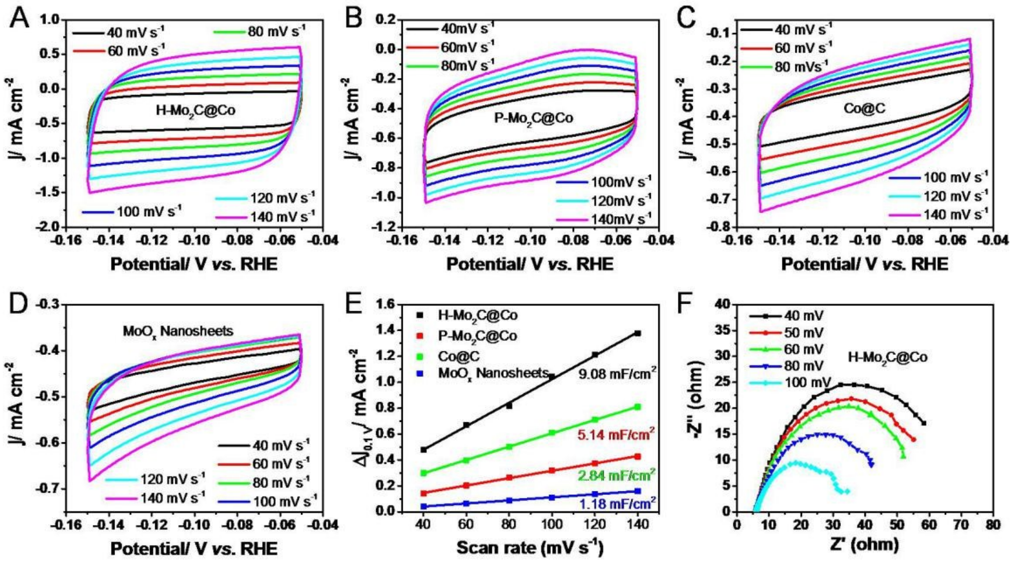

Figure S13. (A-D) Cyclic voltammograms at various scan rates in the region of -0.05V to -0.15 V (vs. RHE) for different catalysts. (E) Variation of double-layer charging currents of different catalysts at 100 mV (vs. RHE) under various scan rates, respectively. (F) Nyquist plots of hierarchical Mo_2C@Co hybrid at various HER overpotentials.

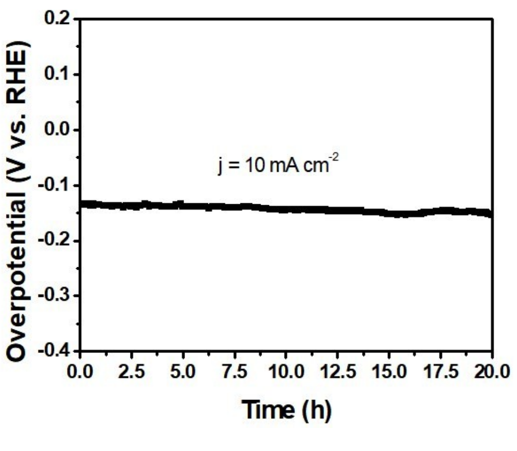

Figure S14. Chronopotentiometric curve recorded at j = −10 mA cm^{-2} for 20 h in 0.5 M H_2SO_4.

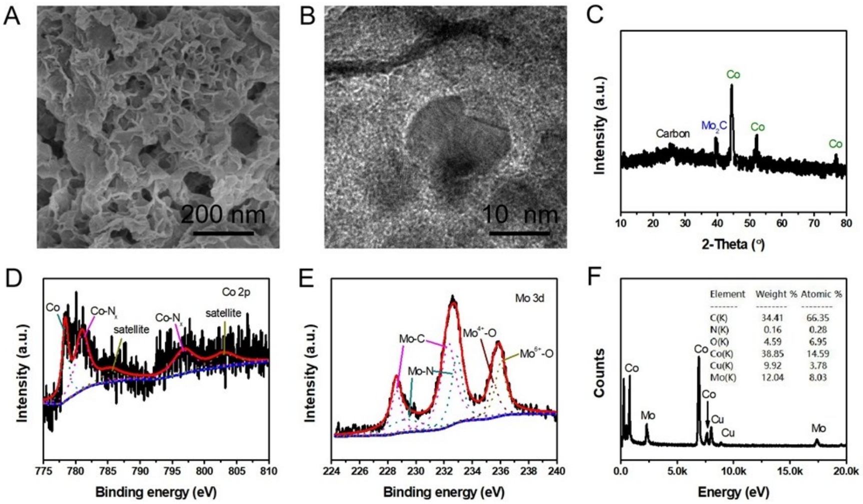

Figure S15. Structure and chemical state analyses of the post-HER H-Mo_2C@Co/CFP electrode after 20 hours' chronopotentiometry test at j = -10 mA cm^{-2}. (A) FESEM, (B) TEM, (C) XRD pattern, (D) Co 2p, (E) Mo 3d core level XPS spectra and (F) EDS , respectively.

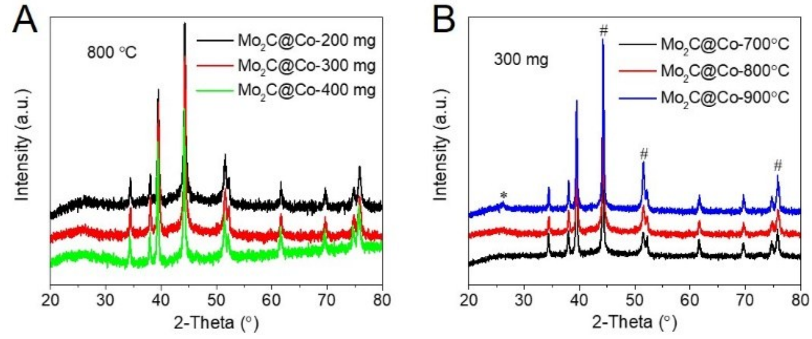

Figure S16. XRD patterns of H-Mo_2C@Co hybrid samples prepared by using different dosage of MoO_x (A) and different annealing temperatures (B).

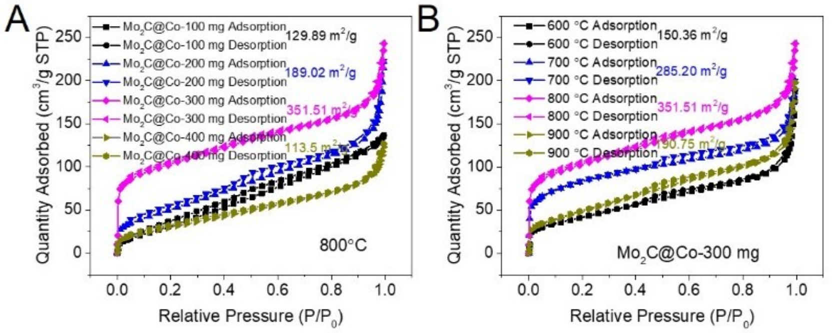

Figure S17. N_2 adsorption-desorption isotherm curves of the different H-Mo_2C@Co hybrid samples. (A) Different amounts of MoO_x nanosheets, (B) Different annealing temperatures.

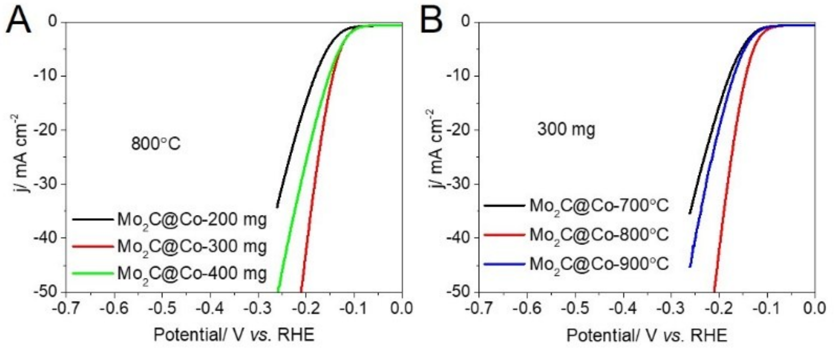

Figure S18. Polarization curves of H-Mo_2C@Co hybrid with different MoO_x dosage and different annealing temperatures in 0.5 M H_2SO_4.

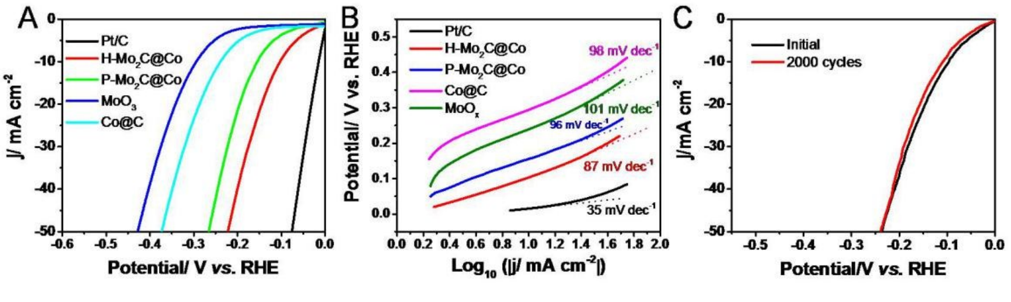

Figure S19. (A) Polarization curves and (B) Tafel plots of H-Mo_2C@Co, P-Mo_2C@Co, MoO_x, Co@C and reference Pt/C, recorded in 1.0 M KOH at a scan rate

of 5 mV s^{-1}. (C) Durability test of H-Mo_2C@Co hybrid in 1.0 M KOH.

Table S1. Comparison of HER performance in acidic and alkaline media of H-Mo_2C@Co with other non-noble metal electrocatalysts.

Catalystl	Current density (j, mA/cm^2)	η at the correspondi ng j (mV)	Tafel slope (mV/dec)	Electrolyte	Reference
H-Mo_2C@Co	10	144	58	0.5 M H_2SO_4	This work
	10	103	87	1.0 M KOH	This work
Ni-Mo_2C/NCNFs	10	196	77.4	1.0 M KOH	Adv. Energy Mater. 2019, 9, 1803185
Co/β-Mo_2C@N-CNTs	10	170	92	1.0 M KOH	Angew. Chem. Int. Ed. 2019, 58, 4923-4928
Ni/Mo_2C-PC	10	210	140	1.0 M KOH	Chem. Sci., 2017, 8 , 968
Ni-Mo_2C@C	10	169	100	0.5 M H_2SO_4	J. Mater. Chem. A 2017, 5, 5000
	10	181	84	1.0 M KOH	
β-Mo_2C nanotubes	10	172	62	0.5 M H_2SO_4	Angew. Chem. Int. Ed. 2015, 54, 15395
	10	112	55	1.0 M KOH	
Mo_2N-Mo_2C/HGr	10	157	55	0.5 M H_2SO_4	Adv. Mater. 2018, 30, 1704156
	10	154	68	1.0 M KOH	
Mo_2C/Graphene composites	10	150	57	0.5 M H_2SO_4	Chem. Commun. 2015, 51, 8323
Mo_2C/CNT-GR	10	148	58	0.5 M H_2SO_4	ACS Nano 2014, 8, 5164
Mo_2C/NCF	10	144	55	0.5 M H_2SO_4	ACS Nano 2016, 10, 11337
Mo_2C/C hybrids	10	146	60	0.5 M H_2SO_4	Adv. Energy Mater. 2017, 7, 1602782
	10	140	58	1.0 M KOH	
Mo_2C@C nano-spheres	10	141	56	0.5 M H_2SO_4	ACS Nano 2016, 10, 8851
Mo_2C/CNT	10	152	65	0.5 M H_2SO_4	Energy Environ. Sci. 2013, 6, 943
Mo_2C/GCSs	10	200	62.6	0.5 M H_2SO_4	ACS Catal. 2014, 4, 2658
MoC_x nano-octahedrons	10	142	53	0.5 M H_2SO_4	Nat. Commun. 2015, 6, 6512
	10	151	59	1.0 M KOH	
Co-Mo_2C@NGCS	10	161	60	1.0 M KOH	Mater. Chem. Front. 2020, 4, 546
CoS-MoS	10	134	59.5	0.5 M H_2SO_4	Adv. Sci. 2019, 6, 1900140
Co_4Mo_2@NC	10	218	74	1.0 M KOH	J. Mater. Chem. A, 2017, 5, 16929
Co-Mo_2C@NCNT	10	186	79	1.0 M KOH	ACS Sustainable Chem. Eng., 2018, 6, 9912-9920.
Mo/Co@N-C	10	187	82	0.5 M H_2SO_4	Small 2018, 14, 1704227
	10	157	148	1.0 M KOH	

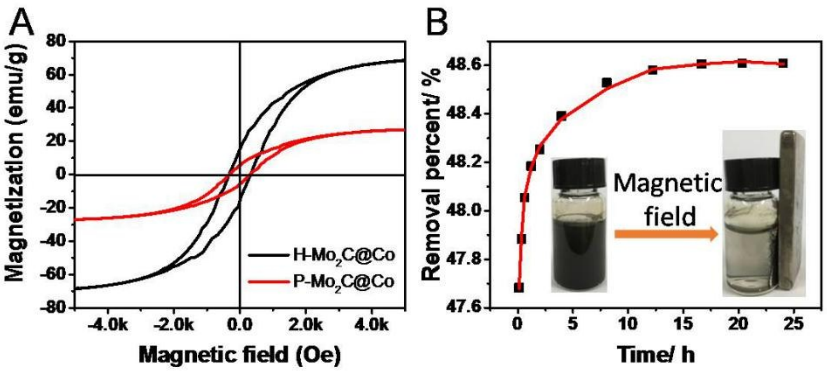

Figure S20. (A) Room-temperature magnetic hysteresis loops of the H-Mo_2C@Co and P-Mo_2C@Co. (B) Time profile of arsenic ion adsorption on H-Mo_2C@Co. The inset shows the H-Mo_2C@Co lifted by a magnet.

Kinetics adsorption of As (V) on H-Mo_2C@Co was investigated in the presence of 0.5 mg mL^{-1} of adsorbent. Figure S15B shows that the adsorption rate is considerably fast within the first 2.5 h, it becomes slow from 2.5 to 12.5 h, and no significant change is observed from 12.5 to 24 h. This phenomenon that the adsorption reached equilibrium state quickly indicates that the adsorption of As (V) on H-Mo_2C@Co was mainly attributed to chemical sorption or surface complexation. The binding of As (V) anions on the surface of H-Mo_2C@Co is ascribed to the strong electrostatic interaction between As (V) and H-Mo_2C@Co structure. In addition, amorphous carbon may provide extra adsorption sites for As adsorption.
